# Patterns and Drivers of Emigration of the Turkish Second Generation in the Netherlands

**DOI:** 10.1007/s10680-021-09598-w

**Published:** 2021-11-22

**Authors:** Petra Wieke de Jong

**Affiliations:** Netherlands Interdisciplinary Demographic Institute (NIDI)-KNAW/University of Groningen, The Hague, The Netherlands

**Keywords:** Emigration, Second generation, The Netherlands, Turkey, Europe

## Abstract

Using unique longitudinal data from the Dutch population registers, this study investigates the patterns and drivers of emigration of the Turkish second generation born in the Netherlands between 1983 and 1992. Around 13% of the Turkish second generation in the research population emigrated during early adulthood, as compared to 6% of their peers without immigrant parents. Half of the Turkish second-generation emigrants who reported their destination country moved to Turkey, while the other half moved to other destinations, especially the Dutch neighbouring countries. Among the Turkish second generation, unemployment over the previous year was found to increase the likelihood of emigration for individuals with low or middle levels of education, whereas no support was found that higher educated individuals (either employed or unemployed) are more likely to emigrate. However, *if* high-skilled unemployed individuals of the Turkish second generation emigrated, they appeared more likely to select Turkey as their destination as compared to other (or unknown) destinations. International migration experiences during childhood, living at the parental home, and residing in neighbourhoods with a high share of co-ethnics were also associated with a higher chance of emigration to Turkey, whereas living in the Dutch border regions was associated with a higher chance of emigration to other destinations. Together, the findings indicate that the Turkish second generation has a higher chance to emigrate than their peers without immigrant parents, and that mechanisms specific to the second generation apply to the migration behaviour of this group.

## Introduction

As a growing number of children of immigrants in Western European countries has become of age over the past decade, the scientific literature has increasingly addressed the transnational behaviour of this so-called second generation. One branch of transnational research focuses specifically on the desire of the second generation to move to the country of origin of their parents (Bettin et al., 2018; Fokkema, 2011; Grasmuck & Hinze, 2016; Groenewold & De Valk, 2017; King & Christou, 2014; Potter, 2005; Tezcan, 2019). This literature on the ‘return intentions’ of the second generation mainly covers the Turkish second generation, who form the largest group with a second-generation migration background in Western European societies. In survey data of the German Socio-economic Panel (GSOEP) and The Integration of the European Second Generation (TIES) project, a significant share of the Turkish second generation expressed at least to some extent the intention to live in Turkey in the future: depending on the national context, shares ranged from 30 to nearly 70% (see Bettin et al., 2018; Groenewold & De Valk, 2017).

In line with this literature on migration intentions, national media in Western European countries over the past decades repeatedly paid attention to the emigration of the Turkish second generation to Turkey.^1^ In these articles, the emigration of this group is often presented as a form of brain drain, caused by economic difficulties and socio-political tensions in the Western European societies these individuals are born and raised in. In light of declining working-age populations in Europe, the emigration of well-educated members of the second generation has become a concern of policymakers as well (Groenewold & De Valk, 2017). Yet while public and political debates assume that the Turkish second generation will move to the country of origin of their parents, the scientific literature has been largely unable to answer questions on whether and where the second generation actually migrates.

With this study, I aim to answer whether the Turkish second generation indeed emigrates more often than their peers without a migration background, where they move to, and what types of factors drive their moves. For this, I use full population register data that captures emigration during early adulthood of the Turkish second generation born in the Netherlands between 1983 and 1992, and a reference group without immigrant parents. The study contributes to the literature in three ways. First, using longitudinal data from the Dutch population registers, this study is among the first to investigate actual emigration behaviour of the second generation as opposed to migration intentions. A focus on the Turkish second generation allows to consider the emigration rates from this study in the light of the literature on migration intentions that also focused primarily on the Turkish second generation. Second, by combining findings from research on transnational behaviour to theory on international migration, the study gains innovative insight into the mechanisms that drive the emigration of the second generation. Specifically, the study addresses whether the chance to emigrate is influenced by the same factors among the second generation as for peers without immigrant parents, or whether mechanisms specific to the second generation occur. Third, while the literature on ‘return intentions’ has focused on desires of the second generation to move to the country of origin of the parents, I broaden the scope of this study to include moves of the second generation directed at other destinations. This way, the study better addresses the need to treat the second generation not just as the descendants of immigrants, but also as full members of their national and European society (Barwick, 2018).

The remainder of this article is structured as follows. In Sect. 2, I continue with a presentation of my theoretical considerations and hypotheses, before providing an overview of the data, operationalization and methods in Sect. 3. Section 4 presents the descriptive findings and results of the analytical models. In Sect. 5, I reflect on the main outcomes and limitations and discuss pathways for future research.

## Theory

### General Determinants of Emigration

Theoretical expectations and empirical findings that specifically address the emigration of individuals with a second-generation migration background are scarce. However, the broader literature on international migration has already identified several social, economic, and geographical factors as important predictors of the chance of emigration. A relevant first step in explaining the emigration behaviour of the Turkish second generation is therefore to investigate whether similar mechanisms are at play among the Turkish second generation and individuals without immigrant parents, and whether differences in emigration rates between the two groups remain when taking their social, economic, and geographical characteristics into account.

#### Social Ties

The international migration literature generally acknowledges that migration decisions are embedded in other life choices, such as career decisions and family formation (Castro-Martín & Cortina, 2015; Dustmann, 2003). To understand which factors increase the chance of emigration, migration scholars therefore often consider indicators in both the public and private domain. Employment status and educational attainment provide insight into the level of economic integration, while partnership status and parenthood are important indicators of an individual’s social commitment to the country of residence (Weber & Saarela, 2019).

To start with the latter, close personal relationships have been found to attach people to the places they live in and tend to prevent them from moving abroad (e.g. Cairns & Smyth, 2011). First and foremost, research has shown that living together with a partner or children typically decreases the preparedness to migrate across borders, as the decision to emigrate will affect the entire household (Dustmann et al., 1996).

**H1** Individuals who have a partner and/or children in the Netherlands are less likely to emigrate than individuals without these social ties.

While the literature does not offer clear reasons to expect that the likelihood of emigration will differ between men and women, several studies have provided evidence that the timing of family formation varies between men and women of the Turkish second generation (Huschek et al., 2010; van Landschoot et al., 2018). In the analyses, I therefore control for possible gender differences.

#### Occupational Status

Beside social commitment, the migration literature generally expects that emigration is linked to a person’s economic status or labour market position. Specifically, being employed can create a form of attachment to the country of residence, thereby lowering the chance of emigration. In turn, one may expect that individuals who are unemployed will be most likely to emigrate, because of weaker socio-economic ties to the country of residence (e.g. Bettin et al., 2018; Todaro, 1969).

**H2** Individuals who are unemployed or inactive are more likely to emigrate than those who are employed.

Recent studies among Western European majority populations further predicted—and to some extent found—the chance of emigration to be higher among the higher educated, who might value the employment opportunities of an international career (e.g. Übelmesser, 2005; Van Dalen & Henkens, 2013). As a theoretical explanation for such educational gradient in international migration decisions, higher educated individuals may consider migration an investment in their human capital, which could generate higher labour market returns (Becker, 1975; Sjaastad, 1962).

**H3** Individuals who have completed higher levels of education are more likely to emigrate than those who completed lower levels of education.

#### Geographical Location

Besides a person’s social and economic position in society, geographical location may shape emigration decisions. First, the big cities of a country often have an important role as stepping stones in international migration processes; a pattern which has been recognized in the migration literature as one of the ‘laws’ of migration (Lee, 1966). Second, living near a country border may facilitate emigration to the neighbouring country, as the costs of international moves are generally lower over short distances (Mayda, 2010). Specific to the Netherlands, prior research has indeed shown that individuals living in the Dutch border regions more often engage in a transnational lifestyle (Gielis, 2009), and that international moves from these regions to the neighbouring countries are quite common due to lower costs of living in Germany and Belgium (Graef & Mulder, 2003; Van Houtum & Gielis, 2006).

**H4** Individuals living in the border regions or largest cities of the Netherlands are more likely to emigrate than those living in other parts of the country.

Taking these geographical factors into account is especially relevant in the context of this study, since the Turkish population in the Netherlands is partly clustered in the border regions of the country—especially the Dutch Provinces Overijssel (bordering Germany) and Brabant (bordering Belgium) where many Turkish first-generation immigrants worked in the textile industry (De Valk et al., 2001)—and partly in the largest cities of the Netherlands (Sleutjes et al., 2018).

### Mechanisms Specific to the Second Generation

Besides these general predictors of the likelihood of emigration, the literature on transnational behaviour points at mechanisms that may be specific to the second generation. Scholars signalled that the second generation faces a rather unique context in which they grow up (Lelie et al., 2012). On the one hand, children of immigrants are brought up in families where the culture and country of origin of their parents typically remain of great importance (Levitt, 2009). The language of their parents is often spoken at home, and ties with family and friends abroad are usually maintained through regular contact and visits. Furthermore, the Turkish second generation often grows up in neighbourhoods with relatively high shares of individuals who share the same ethnic background (Fleischmann et al., 2011), and a strong orientation towards their co-ethnic community (Crul & Doomernik, 2003). When deciding on emigration, the second generation may therefore consider the country of origin of their parents as possible destination country; an option that distinguishes them from individuals without a migration background. At the same time, and in contrast to their first-generation parents, the second generation is born and raised in a European country. Thus, whereas the first generation has clear roots in their country of origin, it seems questionable whether the second generation experiences the same level of connectedness to this country (Alba & Nee, 2003). What is more, born within Europe, the second generation can—like other Europeans—benefit from freedom of movement within the European Union (EU). Second-generation emigrants may therefore also select other destinations than the country of origin of their parents (Barwick, 2018).

#### Cultural and Transnational Factors

Related to this unique position, different cultural or transnational factors may determine the locational choices of second-generation emigrants. To start with, migration experiences during childhood may indicate stronger transnational ties with Turkey. After all, as moves during childhood are typically undertaken together with the parents, it can be expected that individuals of the Turkish second generation who migrated before turning 18 years old spent part of their youth in Turkey. Indeed, findings from a previous study have shown that it is not uncommon for the Turkish second generation to start their educational career in Europe (in this case Belgium), but to subsequently have study periods in Turkey (Neels, 2000). Transnational ties with Turkey may further be stronger for individuals of the second generation who still live with their parents. On a daily basis, these individuals likely experience more of the Turkish language and culture as compared with individuals who already moved out of the parental home (see Extra & Yagmur, 2010; Schneider et al., 2012). For similar reasons, stronger transnational ties can be expected for those who live in neighbourhoods with a higher share of Turkish neighbours. Each of these circumstances increases the likelihood that an individual has connections in Turkey, is proficient in the Turkish language and develops a Turkish cultural identity; factors for which previous studies found a clear link to a higher likelihood of migration intentions to Turkey (e.g. Groenewold & De Valk, 2017).

**H5** Among the Turkish second generation, the likelihood of emigration to Turkey rather than staying in the Netherlands or emigrating elsewhere is higher among individuals who migrated during childhood, those who live at their parental home, and those residing in neighbourhoods with higher shares of Turkish residents.

#### Economic Opportunities

Finally, prior research has often explained the aspirations of the Turkish second generation to migrate to Turkey as “reactive transnationalism”, fueled by experiences of discrimination and lack of opportunities in European societies. According to these studies, cumulative disadvantage can intensify self-identification with the ancestral home country, and in turn increases the likelihood of migration intentions (Groenewold & De Valk, 2017; Tezcan, 2019). Especially high-skilled members of the second-generation may be vulnerable to exclusion when faced with disappointing opportunities considering their qualifications (Fokkema, 2011; Waldinger & Perlmann, 1998). In fact, recent studies revealed that more-educated individuals among the second generation often report higher levels of perceived discrimination—also referred to as ‘integration paradox’ (e.g. de Vroome et al., 2014; Verkuyten, 2016). In turn, one could especially expect unemployed high-skilled individuals to emigrate to Turkey in order to reach the top of their profession.

**H6** Among the Turkish second generation, the likelihood of emigration to Turkey rather than staying in the Netherlands or emigrating elsewhere is higher among unemployed individuals who have completed a higher level of education.

## Data and Methods

### Research Population

The Netherlands is populated by around 17 million people, of whom around 400 thousand have a Turkish background (StatLine, 2019). Since 2013, the second generation makes up more than half of this group. For this study, I am specifically interested in the emigration of the Turkish second generation at adult ages. This because international moves that occur during childhood are likely initiated by the parents, whose underage children have little choice but to join them. As such, emigration during childhood provides less insight into the emigration decisions of the second generation themselves. Therefore, I start my analyses from the moment an individual turns 18 years old, which is the legally defined threshold for adulthood in the Netherlands. At this age, control and legal responsibilities of parents or guardians over their children formally end. Still, it is possible that part of the individuals emigrating at age 18 years old and onwards—although legally independent—moves together with their parents. The data do not permit to rule this out completely. To capture the emigration decisions over early adulthood, individuals in the study population are followed in the analyses until they are at most 35 years old.

### Data

To test my hypotheses, I make use of longitudinal, full population register data from the System of Social Statistical Datasets (SSD) compiled by Statistics Netherlands. The SSD consists of several registers that have been linked to the Dutch municipal population registers (Bakker et al., 2014). For every official inhabitant of the Netherlands, the SSD covers a broad range of demographic and socio-economic indicators, including age, position on the labour market, household composition, and migration. The data cover the full Turkish second generation born between 1983 and 1992, as well as a 10% random sample of all individuals born in the Netherlands over this period without immigrant parents. The latter form the reference group. As the data allow me to follow the migration behaviour until the end of 2017, individuals in the dataset were between 25 and 35 years old at the end of the observation period. In line with the official Dutch statistics, the second generation is defined as individuals who are born in the Netherlands to at least one foreign-born parent. In case both parents are born outside the Netherlands but in different countries, the country of the mother is decisive. However, as the share of mixed marriages is very low among the Turkish first generation, almost all individuals in the study have two parents who were born in Turkey (94%). As I am interested in the emigration of the second generation from 18 years old and onwards, I exclude individuals who lived abroad while turning 18. This results in a study population of around 52,500 individuals with a Turkish second-generation background, and a reference group of around 152,500 individuals born in the Netherlands without immigrant parents.

### Variables

#### Dependent Variable

In the Netherlands, residents are expected to officially deregister at a Dutch municipality if they expect to live outside the Netherlands for at least eight months.^2^ In these cases, the population registers contain reported information on the precise timing of emigration as well as the country of destination. Yet in practice, not everyone who leaves the country to live elsewhere actively deregisters at their Dutch municipality. In this study, I therefore include an additional measure of emigration based on administrative removals from the registers which assumes that individuals whose whereabouts are unknown to the Dutch authorities for more than twelve months have left the Netherlands. Although it seems safe to assume that most individuals without a formal address for more than a year have actually left the Netherlands, one cannot be completely sure that emigration took place in case of these administrative corrections (Alders & Nicolaas, 2003). In addition, because information on the country of destination normally enters the population registers as reported by emigrants when they deregister at a Dutch municipality, information on the country of destination is systematically missing for individuals who emigrate without informing the Dutch authorities. In the separate analyses by destination country, I therefore treat these unregistered moves as a separate group, together with emigrants did who formally deregister but whose destination country is nevertheless unknown.

#### Independent Variables

In the models, I track the household situation and occupational status of the study population by means of time-varying variables. This way, important life course transitions that often occur over early adulthood, like leaving the parental home, getting married, having a child or entering the labour market, are captured in the models in a dynamic way. Regarding household composition, the time-varying variables *partner* and *child* indicate for each year since turning 18 whether an individual is married or in a registered partnership, and has a child or not (1 = yes, 0 = no). The variable *parental home* indicates for each year whether an individual is registered to live with his or her parents (1 = yes, 0 = no). Regarding socio-economic position, I consider a person’s main occupation together with one’s highest obtained level of education. This because the impact of being unemployed on emigration behaviour may vary depending on the (perceived) chances of employment abroad, which likely vary by level of education. Instead of entering main occupation and highest level of education into the model as two separate variables, I therefore combine the dimensions into a single time-varying variable, distinguishing the following five categories: (1) Employed, completed low or middle levels of education; (2) Employed, completed tertiary levels of education; (3) Enrolled in education; (4) Unemployed, completed low or middle levels of education; (5) Unemployed, completed tertiary levels of education.^3^ As indicator of *childhood migration experience*, I capture whether a person—according to the Dutch population registers—ever emigrated from the Netherlands before turning 18 years old (1 = yes, 0 = no). Thus, individuals with a childhood migration experience are individuals who are born in the Netherlands, who lived at least eight months of their childhood in another country, and who returned to the Netherlands before turning 18 years old.^4^ Of the Turkish second generation with such a childhood migration experience, more than 90% moved to Turkey during childhood. This figure supports my argumentation that emigration during childhood for the Turkish second generation often signals that these individuals spent part of their childhood in the country of origin of their parents, which may lead to stronger ties with Turkey. Specific for the Turkish second generation, the variable *co-ethnic neighbourhood* distinguishes neighbourhoods in which individuals with a Turkish background (first- and second generation) made up at least 15 percentage of the population (1 = 15% or more, 0 = less than 15%). This threshold distinguishes the quartile of the second generation in the research population living in neighbourhoods with the highest shares of Turkish neighbours from neighbourhoods with lower shares. The dummy variable *male* (1 = male; 0 = female) controls for differences between men and women. The variable *border region* captures whether a person is living in one of the border regions that connect the Netherlands to Germany or Belgium (1 = yes, 0 = no). I define border regions as the regions at NUTS 3 level (or ‘COROP regions’) located directly at the German or Belgian border.^5^ The variable *population density* identifies residents of the most densely populated areas of the Netherlands (1 = more than 2500 households per km^2^, 0 = 2500 or less households per km^2^).

For a small share of the person years (0.1%), data on main occupation were missing. Individuals who are formally employed or enrolled in education are more likely to be registered as such in the population registers, as these statuses are linked to formal arrangements at the institutional level (i.e. the employer or educational institution). For this substantive reason, I grouped missing cases with category 3 = ‘unemployed or inactive’. Furthermore, for less than 1 percentage of the observations (0.2%), information on the place of residence was missing. For these cases, I imputed *co-ethnic neighbourhood* with value 0 = ‘percentages less than 15%’; *border region* with value 0 = ‘not living in a border region’ and *population density* with value 0 = ‘less than 2500 addresses per square kilometre’, for these variables to underestimate rather than to overestimate the impact of these dimensions.

### Methodology

To investigate the emigration of the Turkish second generation, I use an event history setup. This approach allows to analyse the time to an event (in this case emigration) and is less sensitive to right-censoring or skewed distributions in the dependent variable than alternative estimation methods, like logistic and multinomial regression models (Allison, 2014). What is more, event history models better allow to capture crucial transitions in the life course that often occur over early adulthood, like leaving the parental home, having a child or the shift from education to entering the labour market. This as opposed to cross-sectional models which simply control for a person’s occupational status and household conditions at the moment of emigration. In a first, more descriptive part of the analyses, I present the prevalence of emigration of the Turkish second generation and their peers without a migration background. To test my expectations on the drivers of emigration, I subsequently estimate event history models for the two groups separately. In a final step, and specific for the Turkish second generation, I compare the drivers of emigration to Turkey and emigration to other destinations by means of a multinomial event history model. In this final model, the likelihood of emigration to Turkey, emigration to an alternative destination and emigration to an unknown destination are contrasted with the likelihood of staying in the Netherlands. Findings from all event history models are reported with robust standard errors, which correct for the clustering of person years within individuals. In the first analyses, I further apply frequency weights to account for the fact that the complete Turkish second generation is included in the data, as compared to a 10% random sample of the majority population.

## Results

### Descriptive Statistics

Of the Turkish second generation in the research population, 87% never emigrated between 2001 and 2017, while around 13% (*N* = 6914) emigrated at least once from the Netherlands at age 18 or higher. As such, the emigration rate was more than twice as high for the Turkish second generation than for their peers without immigrant parents: among the latter group, only 6% (*N* = 9220) emigrated over the observed years. Among the Turkish second generation, around 20% of the emigrants entered the data through administrative corrections, versus around 13% of the emigrants without immigrant parents.

Table 1 presents the destination country of the first emigration since turning 18 years old as reported by emigrants at a Dutch municipality. Around 38% of the Turkish second-generation emigrants selected Turkey as their destination country, while around 37% chose a different destination. For the remaining 25%, the destination was unknown. After Turkey, Belgium (17%) and Germany (12%) were the most frequently selected destinations. Also for Dutch emigrants without a migration background Belgium (14%) and Germany (12%) were the most frequently selected destination countries, followed by the UK (10%). Over 53% of the emigrants without immigrant parents selected a European destination country, versus 34% of the Turkish second generation. Around 5% of the Turkish second-generation emigrants who officially deregistered at a Dutch municipality did not report a country of destination, versus 2% of the emigrants without a migration background.

**Table 1:** Destination of emigrants of the Turkish second generation and a 10% random sample of the majority population

Destination	Turkish second generation		Majority population	
	*N*	%	*N*	%
Turkey	2612	37.78	46	0.50
Belgium	1208	17.47	1286	13.95
Germany	805	11.64	1078	11.69
UK	102	1.48	880	9.54
Other EU country	237	3.43	1686	18.29
Other non-EU country	189	2.73	2882	31.26
Unknown	1761	25.47	1362	1477
Total	*6914*	*100*	*9220*	*100*

A detailed analysis of the factors predicting the likelihood that emigrants at a later stage move back to the Netherlands is beyond the scope and setup of the current study. After all, the data used for this study do not provide insight into whether emigrants planned a short-term or long-term (perhaps even permanent) stay abroad when they left the Netherlands, nor capture changes in partnership, parental or occupational status since leaving the Netherlands. However, it still seems relevant to note that slightly more than half (53%) of all second-generation emigrants who left the Netherlands over the observation period, moved back to the Netherlands before the end of 2017. This share was somewhat lower among emigrants to Turkey (43%) as compared with emigrants to other destinations (47%). Of the emigrants who did not report their country of destination, around two third (74%) resided in the Netherlands again at the end of the observation period. Of the emigrants without immigrant parents, 59% moved back to the Netherlands over the observed years.

### Incidence of Emigration

**Fig. 1:**
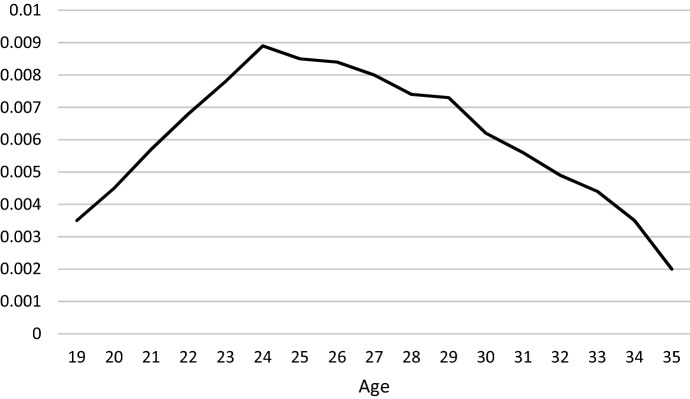
Hazard rate for emigration

**Fig. 2:**
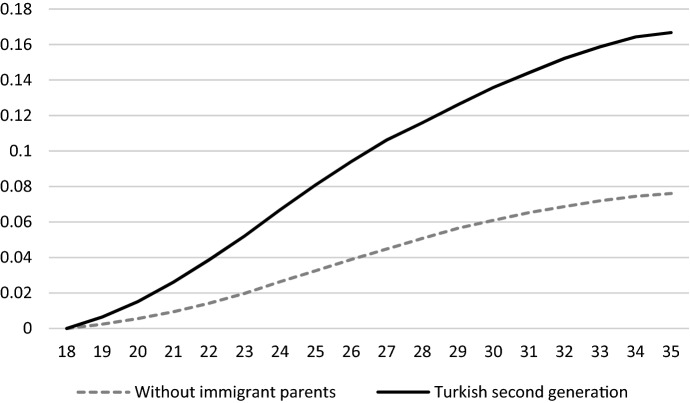
Survival estimates for emigration by migration background

Figure 1 presents the prevalence of emigration for the second generation and their peers without immigrant parents since turning 18 years old. The figure shows that the hazard of emigration peaks at age 24 and decreases with higher ages. In other words, those individuals who emigrate during early adulthood are most likely to do so at relatively young ages. Figure 2 presents the survival estimates for emigration of the Turkish second generation and their peers without immigrant parents separately. The process time for emigration starts at age 18, and the population at risk consists of all individuals within the study population. Individuals are right censored at the end of the observation period in December 2017. The risk of emigration over early adulthood is clearly higher for the Turkish second generation as compared to their peers without immigrant parents.

### Results from the Event History Models

In line with the descriptive statistics, the first event history model (column 1, Table 2) shows a higher chance of emigration for the Turkish second generation as compared to peers without a migration background. However, differences in emigration rates between the Turkish second generation and individuals without immigrant parents may follow primarily from these groups holding different positions in Western European societies. To account for potential composition effects, the second model (column 2, Table 2) therefore includes all explanatory variables. Results of this model show that having a second-generation migration background continues to have a strong and positive impact on the likelihood of emigration after controlling for social, economic and geographical factors. This result possibly indicates that mechanisms specific to the second generation are at play. However, it is also possible that still other general mechanisms are at play that may explain the differences between the two groups, yet that go unobserved in the present model.

**Table 2:** Event history models predicting emigration, ref. staying in the Netherlands

	Model 1		Model 2	
	OR	SE	OR	SE
Time	1.29***	0.01	1.24***	0.01
Time squared	0.98***	0.00	0.99***	0.00
Second generation	2.37***	0.04	1.99***	0.04
Partner			0.66***	0.03
Children			0.39***	0.02
*Socio-economic status (ref. Employed, low/middle education)*				
Employed, higher education			0.70***	0.03
Enrolled in education			1.22***	0.03
Unemployed, low/middle education			2.10***	0.07
Unemployed, higher education			2.25***	0.16
Parental home			0.69***	0.02
Childhood migration experience			3.92***	0.23
Border region			1.20***	0.03
Urban area			1.50***	0.03
Male			0.96	0.02
Constant	0.00***	0.00	0.00***	0.00
*N person years*	*18,931,875*		*18,931,875*	
*N individuals*	*205,074*		*205,074*	

*Significant at the 5% level

**Significant at the 1% level

***Significant at the 0.1% level

#### Separate Models by Migration Background

To investigate whether the same mechanisms explain the likelihood of emigration among the Turkish second generation and individuals without immigrant parents, Table 3 presents the results of the full model separately for the two groups. In line with H1, the risk of emigration is lower among individuals who have a partner as well as those who have children for both the Turkish second generation and the group without immigrant parents. In contrast, the effects of socio-economic status appear to vary between the Turkish second generation and individuals without immigrant parents. Recall that I expected individuals who are unemployed to be more likely to emigrate than those who are employed (H2), and individuals with higher levels of completed education to be more likely to emigrate than those with lower levels of education (H3). Among the group without immigrant parents, the chance of emigration is indeed highest for individuals who were unemployed over the previous year, in particular those who completed higher levels of education, as proposed by H2 and H3. Among the Turkish second generation, the likelihood of emigration is highest among the unemployed as well, yet only for those who completed low or middle levels of education. As a further difference, being enrolled in education lowers the chance of emigration among the Turkish second generation, whereas the opposite holds true for those without immigrant parents. For both groups, the likelihood of emigration is lowest among the employed who completed tertiary education. Thus, among the Turkish second generation, the findings support the hypothesis that unemployment increases the likelihood of emigration for individuals with low or middle levels of education, whereas no support is found that higher educated individuals (either employed or unemployed) are more likely to emigrate.

**Table 3:** Event history models predicting emigration of the Turkish second generation and the majority population, ref. staying in the Netherlands

	Emigration		Emigration	
	Turkish second generation		Majority population	
	OR	SE	OR	SE
Time	1.14***	0.01	1.24***	0.01
Time squared	0.99***	0.00	0.99***	0.00
Partner	0.47***	0.02	0.68***	0.04
Child	0.78***	0.04	0.37***	0.02
*Socio-economic status (ref. Employed, low/middle education)*				
Employed, higher education	0.47***	0.04	0.70***	0.03
Enrolled in education	0.61***	0.02	1.25***	0.04
Unemployed, low/middle education	1.56***	0.05	2.13***	0.07
Unemployed, higher education	1.02	0.13	2.31***	0.17
Parental home	0.86***	0.02	0.68***	0.02
Border region	1.32***	0.04	1.18***	0.03
Urban area	0.93*	0.03	1.55***	0.04
Childhood migration experience	2.63***	0.14	4.13***	0.27
Male	0.61***	0.02	0.99	0.02
Constant	0.02***	0.00	0.00***	0.00
*N individuals*	*52,509*		*152,565*	
*N person years*	*585,805*		*18,346,070*	

*Significant at the 5% level

**Significant at the 1% level

***Significant at the 0.1% level

Regarding geographical location, I expected individuals living in the border regions or largest cities of the Netherlands to be more likely to emigrate than those living in other parts of the country (H4). In line with this expectation, both factors have a positive and significant impact on the likelihood of emigration among individuals without immigrant parents. However, for the Turkish second generation, H4 is only supported regarding border regions. Living in the most densely populated areas on the other hand has a small *negative* impact on the likelihood of emigration, indicating that among the Turkish second-generation individuals residing in the big cities of the Netherlands are somewhat less likely to emigrate than those living in other parts of the country. Finally, and although no hypotheses were formulated regarding gender differences, men of the Turkish second generation appear less likely to emigrate than women, whereas no significant gender difference occurs among their peers without immigrant parents.

#### Emigration of the Second Generation by Destination

To test the final two hypotheses, Table 4 presents the results of a separate model for the Turkish second generation which compares the drivers of emigration to Turkey with those to alternative destinations. Based on literature on transnationalism, I expected that the likelihood of emigration to Turkey rather than staying in the Netherlands or emigrating elsewhere is higher among those who migrated during childhood, who live at their parental home, or who reside in neighbourhoods with higher shares of Turkish residents (H5). Supporting H5, the results show that emigration during childhood increases the likelihood of emigration in general, but particularly to Turkey. Further in line with H5, the likelihood of emigration to Turkey rather than staying in the Netherlands or emigrating elsewhere is higher for individuals living at the parental home or in neighbourhoods with a high share of Turkish neighbours. Interestingly, residing in a Dutch border region does not affect the likelihood of emigration to Turkey, whereas it has a significant and positive impact on emigration to other destinations. These findings suggest that different contextual factors (i.e. ethnic composition of the neighbourhood, living near a country border) influence the type of transnational ties that an individual develops, and help to understand both the prevalence of emigration and the chosen destination. Interestingly, living in a densely populated area appears to have a negative impact on emigration to Turkey and (to a lesser extent) to other destinations, whereas it has a positive impact on emigration with an unknown destination.

**Table 4:** Multinomial event history models predicting emigration of the Turkish second generation, ref. staying in the Netherlands

	Emigration to		Emigration to		Emigration to	
	Turkey		Other destination		Unknown destination	
	RRR	SE	RRR	SE	RRR	SE
Time	1.26***	0.03	1.15***	0.02	0.99	0.02
Time squared	0.99***	0.00	0.99***	0.00	1.00**	0.00
Partner	0.53***	0.03	0.56***	0.04	0.26***	0.03
Child	0.79***	0.06	0.72***	0.06	0.97	0.10
*Socio-economic status (ref. Employed, low/middle education)*						
Employed, higher education	0.50***	0.06	0.56***	0.06	0.14***	0.05
Enrolled in education	0.75***	0.04	0.65***	0.04	0.40***	0.03
Unemployed, low/middle education	1.60***	0.09	1.00	0.06	2.34***	0.14
Unemployed, higher education	1.53**	0.24	0.62	0.16	0.39	0.19
Co-ethnic neighbourhood	1.22***	0.06	1.00	0.05	1.26***	0.07
Parental home	1.15**	0.05	0.90*	0.04	0.53***	0.03
Border region	1.02	0.05	2.17***	0.1	0.86*	0.06
Urban area	0.78***	0.04	0.89*	0.04	1.20***	0.07
Childhood migration experience	3.68***	0.29	1.80***	0.19	2.32***	0.24
Male	0.31***	0.01	0.55***	0.02	1.84***	0.10
Constant	0.00***	0.00	0.01***	0.00	0.00***	0.00
*N individuals*						*52,509*
*N person years*						*585,805*

*Significant at the 5% level

**Significant at the 1% level

***Significant at the 0.1% level

Finally, H6 expects a higher likelihood of emigration to Turkey as opposed to staying in the Netherlands or emigrating elsewhere among unemployed individuals with higher levels of education. Supporting H6, unemployed emigrants who completed higher levels of education opt more often for Turkey as opposed to other or unknown destinations. Being unemployed over the previous year seems to have less of an effect on the chance of emigration to other destinations (often Germany or Belgium), whereas individuals who did not report their country of destination mainly emigrated from a situation of unemployment with low or middle levels of completed education. However, it should be noted that only a small share of the Turkish second generation in the research population was unemployed and highly educated over the years under observation, and that the likelihood of emigration to Turkey rather than staying or moving elsewhere is still highest for individuals who are unemployed with low or middle levels of education. As a further finding of interest, among the Turkish second generation, individuals who are enrolled in education are less likely to emigrate, regardless of the destination country. This in contrast to their peers without immigrant parents, who seem to emigrate more often as part of their study (see Table 3).

## Discussion

Research on migration intentions has shown that a substantial share of the Turkish second generation in Western European societies considers a future move to their parents’ country of origin. However, in absence of suitable data, the scientific literature has been largely unable to answer whether the second generation actually emigrates more often than their peers without a migration background, and what types of factors drive their moves. Furthermore, by focusing on so-called return intentions, previous studies mainly addressed the kind of transnationalism that connects the country of residence to the ancestral home country. In result, we know little about alternative forms of mobility of the second generation, which cover destinations other than the parents’ country of origin (Barwick, 2018). This is an important shortcoming, especially in the European context, where distances between countries are easy to bridge, and where citizens with a permanent residence status (like the second generation) can move freely between countries in the Schengen area (Atger, 2008). Introducing unique longitudinal data from the Dutch population registers, this study aimed to address these knowledge gaps by investigating the actual migration behaviour of the Turkish second generation. This way, the study provides new insights on whether the second generation actually moves more often than their peers without immigrant parents, where they move to, and what types of factors drive their moves.

Over the observed years, around 13% of the Turkish second generation emigrated from the Netherlands. This share is more than twice as high as compared to their peers without a migration background, of whom around 6% emigrated over this period. However, the share who emigrated over the observation period was clearly lower than the percentage of the Turkish second generation in the Netherlands who said to consider emigration to Turkey in previous research, which was as high as 55% in the study by Groenewold and De Valk (2017). Furthermore, and contrasting popular beliefs, the Turkish second generation does not necessarily emigrate to Turkey. In fact, half of the emigrants who actively deregistered at a Dutch municipality due to emigration selected another destination, especially Germany and Belgium, much like their peers without immigrant parents.

In research on migration intentions among the Turkish second generation, respondents expressing the intention to move to Turkey typically felt more connected to Turkey, and maintained stronger transnational ties through Turkish-language media, regular contact with friends or relatives living in Turkey, as well as longer or more frequent visits (Fokkema, 2011; Groenewold & De Valk, 2017; Tezcan, 2019). In a similar vein, findings of this study show that the Turkish second generation who had a migration experience during childhood, those living at the parental home, and those residing in neighbourhoods with higher shares of co-ethnic neighbours were more likely to emigrate to Turkey rather than staying in the Netherlands or emigrating elsewhere. Especially migration during childhood appeared a strong predictor of the likelihood of emigration, not just for the second generation but also for individuals without immigrant parents (see Table 2). This suggests that international migration during childhood accustoms individuals to moving in a way that raises their likelihood to become internationally mobile later in life. While prior research has investigated the impact of a childhood migration experience on other outcomes, such as socio-economic status (e.g. Neels, 2000) and mental health (e.g. Patterson et al., 2013), I am not aware of any previous work that connects childhood migration experiences to international migration later in life. Further research is therefore needed to investigate the transferability of this finding to other groups and contexts, as well as to shed more light on the underlying mechanisms.

Both the scientific literature and national news media have suggested that especially the highly educated members of the Turkish second generation are likely to become mobile, because of disappointing labour market opportunities in their Western European countries of residence. However, findings of this study do not fully support this expectation. Unemployment over the previous year generally increased the likelihood of emigration among both the Turkish second generation and their peers without immigrant parents. Yet whereas among individuals without immigrant parents the impact of unemployment was strongest for the higher educated, among the Turkish second generation the strongest impact was observed for those with low or middle levels of education. Regarding these findings, one should bear in mind that the data only allow to distinguish individuals who completed tertiary levels of education from those who did not. As children of immigrants often aspire to surpass their parents’ socio-economic status (e.g. Kunuroglu et al., 2018; Schaeffer, 2019), it is possible that among this group especially those individuals with middle levels of education emigrate more often because of disappointing economic opportunities in the country of residence.

Furthermore, when distinguishing emigrants of the second generation by destination country, unemployed individuals with higher education levels appeared more likely to move to Turkey as compared to other destinations. This result may indicate that unemployed, higher educated members of the Turkish second generation perceive better employment opportunities in Turkey as opposed to other destinations, and that *if* they become mobile, they are more likely to emigrate to Turkey. Such findings may distinguish the Turkish second generation from other groups with a non-Western second-generation background, like the Moroccan group. After all, as opposed to Morocco, Turkey experienced substantial economic growth over the past decades, possibly resulting in the country becoming a more attractive destination for the second generation (Çelik & Notten, 2014). Indeed, figures on the countries of destination selected by emigrants from the Netherlands show that the Turkish second generation more often moved to the country of origin of their parents than the Moroccan second generation (de Jong et al., 2020). While incorporating characteristics of the situation in Turkey into the analyses is beyond the scope of the present study, the findings thus emphasize the importance of understanding migration decisions from the interplay between (micro- and macro-level) factors in both the country of origin and destination.

Although I did not formulate specific hypotheses on gender differences, the chance of emigration appeared much lower for men than for women of the Turkish second generation. Such gender differences in the prevalence of emigration may have important consequences for the demographic composition of the Turkish second generation in the Netherlands. Previous research has indicated that women of the Turkish second generation more often than men marry a first-generation Turkish spouse (Huschek et al., 2012; Timmerman et al., 2009). As the Dutch government has implemented regulations that restrict immigration for family formation from Turkey, the results possibly indicate that women of the Turkish second generation now emigrate to Turkey to marry a Turkish spouse. As the register data do not provide information on migration motives, additional research is needed to further investigate this and other possible explanations.

Another important pathway for future research concerns the question to what extent the emigration decisions of the second generation are of permanent or temporary nature. By labelling the desires to move to Turkey as ‘return intentions’, previous studies have implicitly suggested that individuals who express such intentions consider leaving their European country of birth for good. However, while first-generation immigrants typically face legal hurdles to move back and forth, temporary moves are likely easier and more common for their children, who often hold a European passport. Supporting this expectation, in a qualitative study among the Turkish second generation born and raised in Germany yet residing in Turkey at the time of the interview, respondents did not necessarily perceive their move to Turkey as being “forever” (Tılıç-Rittersberger et al., 2011). In fact, many of these individuals had taken dual citizenship before they emigrated to facilitate potential immigration to Germany in the future. Detailed analyses of the temporality of emigration are beyond the scope of this study, as this would either require data that enable researchers to follow persons after emigration, or survey data regarding the intended length of stay at the time of emigration. An example of the former is the study by Weber and Saarela (2019), who studied the emigration and return of Fins to Sweden by linking data from the Finnish and Swedish population registers. Given the availability of high-quality register data in the Netherlands and Belgium, future research could adopt a similar approach to investigate the migration dynamics of the Turkish second generation between these two countries. As knowledge of the intra-European migration behaviour of the second generation is virtually absent, this type of research would provide a valuable addition to the literature on the second generation in European societies.

To conclude, this study is among the first to show that the Turkish second generation is more likely to emigrate over early adulthood than their peers without a migration background. Still, the share who emigrated over the observation period was substantially lower than the share of the Turkish second generation reporting migration intentions in previous studies. Furthermore, the study showed how among those who emigrated, the country of origin of the parents appears just one of the frequently selected destinations, rather than the sole destination. The study places expectations of a ‘brain drain’ of the second generation to the country of origin of their parents in perspective. Transnational ties and geographical location on the other hand are identified as relevant indicators of both the likelihood of emigration and the locational choices of the Turkish second generation. Together, the findings of this study stress the relevance of paying special attention to second generation migrant populations when investigating emigration behaviour in Western European societies.

## Data Availability

The data that support the findings of this study have been prepared for analysis at Statistics Netherlands. The data are not publicly available for privacy reasons.

## Footnotes

1 Trouw, 6 April 2005 ‘Liever terug naar Turkije’; Der Spiegel, 10 September 2009 ‘Exodus von Mustermigranten’; Der Spiegel, 14 September 2010 ‘Rückkehrende Migranten: Nie mehr braver Türke’; De Volkskrant, 21 November 2010 ‘Jongeren terug naar het land van hun Turkse ouders’; NRZ, 16 June 2011 ‘Akademiker türkischer Herkunft kehren Deutschland den Rücken; De Standaard, 23 April 2017, ‘Turken keren België rug toe’.

2 See https://www.cbs.nl/nl-nl/onze-diensten/methoden/begrippen for the definitions of Statistics Netherlands.

3 Information on the highest completed level of education did not directly enter the Dutch population registers until 2006. In result, and while high levels of education have been registered accurately for the years under study, more detailed information on middle and lower levels of education is missing for earlier years.

4 Concerning childhood migration experience, one should be aware that data on migration is included in the Dutch population registers since 1995. Although Statistics Netherlands retrospectively registered part of the international moves prior to 1995, the variable on childhood migration likely underestimates the number of individuals who emigrated before 1995 due to this feature of the data.

5 For the division of the Netherlands in the 40 COROP regions, see https://www.regioatlas.nl/kaarten#_coropregios.
